# 912 Burn Surgery Bibliometric Analysis of Research Funding at American Burn Association-Verified Burn Centers

**DOI:** 10.1093/jbcr/iraf019.443

**Published:** 2025-04-01

**Authors:** Tiffany Jeong, Nicolas Kass, Mare Kaulakis, Christopher Fedor, Hilary Liu, José Arellano, Francesco Egro

**Affiliations:** University of Pittsburgh School of Medicine; University of Pittsburgh Medical Center; University of Pittsburgh School of Medicine; University of Pittsburgh School of Medicine; University of Pittsburgh Medical Center; University of Pittsburgh Medical Center; University of Pittsburgh Medical Center

## Abstract

**Introduction:**

Evidence based surgery and management arises from high quality research that informs practice and policy recommendations. However, burn surgical research is a costly endeavor and the geographical distribution of American Burn Association (ABA)-verified burn centers is unequal. Through comprehensive examination of literature, this article aims to investigate the interplay between research funding, research productivity, and ABA-verification in burn surgery.

**Methods:**

PubMed was queried for all articles relevant to the burn surgery/reconstruction from June 6th, 2024 to January 1st, 1844. Data was pulled on the article title, research funding, article type, and affiliation. American Burn Association-verified burn centers were identified through the ABA website on July 14th, 2024. Affiliated academic institutions for burn centers were identified through Google search.

**Results:**

16138 articles on the subject of burn surgery/reconstruction were identified with PubMed query from June 8th, 2024, to January 1st, 1844. The majority of these were original journal articles (93.6%, n=15113). Overall, 16.6% of articles report funding (n=2671).

Only a minority of studies are considered high level evidence, with 2.1% clinical trials(n=338) and 0.6% metanalyses (n=103). However, high level evidence research was more likely to be funded than other studies (32.4% vs 16.1%, p-value < 2.2e-16). Thus, raising the level of evidence in the field of burn surgery will likely require greater investment from research organizations like the NIH, DOD, and NSF.

When considering the article affiliations, 13.5% (n=2172) originated for American Burn Association-verified medical centers. Affiliation with an ABA-verified burn center was significantly associated with having research support (30.5% vs 14.4%, p-value < 2.2e-16), suggesting that higher standards in burn care fosters high-quality funded literature. The most prolific ABA-verified burn centers were Shriners Children’s Hosptial-Boston/Brigham and Women’s Hospital/Harvard (n=379), Blocker Burn Unit/UTMB (n=377), and Johns Hopkins Adult/Pediatric Burn Center (n=158).

**Conclusions:**

High level evidence comprises a minority of articles in burn surgery literature but are significantly associated with research funding. Furthermore, ABA-verified burn centers have a higher percentage of their research funded. While we are unable to elucidate whether ABA verification or research funding drives these phenomenon, it remains clear that high level burn surgery research, funding, and ABA-verification often coexist.

**Applicability of Research to Practice:**

Researchers interested in elevating the quality of their research or funding may consider verification through the ABA at their burn center. Furthermore, this research may point to the need for pilot grants or other funding at smaller institutions that are not ABA verified.

**Funding for the Study:**

N/A

